# Efficacy and safety of minimally invasive percutaneous nephrolithotomy versus retrograde intrarenal surgery in the treatment of upper urinary tract stones (> 1 cm): a systematic review and meta-analysis of 18 randomized controlled trials

**DOI:** 10.1186/s12894-023-01341-3

**Published:** 2023-10-24

**Authors:** Yang Liu, Huimin Zhang, Zhi Wen, Yu Jiang, Jing Huang, Chongjian Wang, Caixia Chen, Jiahao Wang, Erhao Bao, Xuesong Yang

**Affiliations:** 1 Department of Urology, Affiliated Hospital of North Sichuan Medical College, Nanchong, China; 2 Department of Urology, Chengdu Xinhua Hospital Affiliated to North Sichuan Medical College, ChengDu, China; 3 Department of Radiology, Affiliated Hospital of North Sichuan Medical College, Nanchong, China

**Keywords:** mPCNL, RIRS, Urinary tract stones, Stone free rate, Urolithiasis, Complications

## Abstract

**Background:**

The advantages and disadvantages of retrograde intrarenal surgery (RIRS) and minimally invasive percutaneous nephrolithotomy (mPCNL) for treatment of upper urinary tract calculi have not been conclusively determined.

**Methods:**

In this meta-analysis, We comprehensively evaluated the performance of the two surgical approaches in treatment of upper urinary calculi. We searched the Pubmed, Embase, Cochrane and Web of science databases for randomized controlled trial (RCT) articles on RIRS and mPCNL upto December 2022. Data were extracted by two independent reviewers and subjected to the meta-analysis using the Stata 15.1 software (StataSE, USA).

**Results:**

A total of 18 eligible RCTs involving 1733 patients were included in this study. The meta-analysis revealed that mPCNL of 1–2 cm or 2–3 cm stones had a higher stone clearance rate (RR:1.08, 95%CI (1.03, 1.14), *p* = 0.002) and shorter operation time (WMD : -10.85 min, 95%CI (-16.76, -4.94), *p*<0.001). However, it was associated with more hospital stay time (WMD :1.01 day, 95%CI(0.53, 1.5), *p*<0.001), hemoglobin drops (WMD :0.27 g/dl, 95%CI (0.14, 0.41), *p*<0.001), blood transfusion rate (RR:5.04, 95%CI(1.62, 15.65), *p* = 0.005), pain visual analogue score (WMD:0.75, 95%CI (0.04, 1.46), *p* = 0.037), hospital costs (SMD :-0.97, 95%CI (-1.19, -0.76), *p*<0.001) and major complications (RR:1.89, 95%CI(1.01, 3.53), *p* = 0.045).

**Conclusion:**

Therefore, in terms of surgical effects and operation time, mPCNL is superior to RIRS, but is inferior with regards to other perioperative parameters. These factors should be fully considered in clinical decision making.

**Supplementary Information:**

The online version contains supplementary material available at 10.1186/s12894-023-01341-3.

## Introduction

Globally, the incidences and recurrence rates of kidney stones are [1–3], resulting in a significant increase in treatment costs and substantial health challenges. Regarding treatment, most patients with stones < 1 cm will pass the stones automatically after analgesia and oral stone lysis, but patients with stones > 1 cm usually require urological interventions [4, 5]. The current mainstream methods of surgical interventions include retrograde intrarenal surgery (RIRS), standard percutaneous nephrolithotomy (sPCNL) and minimally invasive or Micro or Ultra-mini or super-mini percutaneous nephrolithotomy (mPCNL). Retrograde intrarenal surgery is suitable for stones less than 2 cm while percutaneous nephrolithotomy is suitable for stones more than 2 cm [6, 7]. Due to advances in laser and surgical technologies, minimally invasive percutaneous nephrolithotomy has attracted people’s attention because of its lower complications and higher stone clearance rate when compared with standard percutaneous nephrolithotomy [8–11].

Previously, meta-analyses have compared the outcomes of the two procedural approaches, asserting that mPCNL yields superior surgical outcomes compared to RIRS [12–16]. However, their conclusions regarding postoperative complications and related aspects are conflicting. Constrained by the quality and sample size of the included studies, prior research findings necessitate validation through prospective large-scale investigations. In recent years, a plethora of well-designed Randomized Controlled Trials (RCTs) addressing this subject have been published; nevertheless, a consensus on the conclusions has yet to be reached [17–21].

Henceforth, we aim to incorporate and synthesize the most recent RCT publications, with the intention of furnishing a higher level of evidence for the comparative efficacy and safety of mPCNL and RIRS interventions in the treatment of renal stones exceeding 1 cm. This endeavor seeks to underpin clinical decision-making processes.

## Methods

This systematic review and meta-analysis was conducted following the methods recommended by the Preferred Reporting Project for Systematic Reviews and Meta-analyses (PRISMA) statement [22] and registered on the Prospero website (CRD42023387706).

### Literature search, inclusion, and exclusion criteria

The Pubmed, Cochrane Library, Web of Science, and Embase databases were searched for published articles until December 20, 2022. The search was carried out using a combination of subject headings and free words. The following search strategy was developed on the basis of Intervention and patient-related characteristics: ((Kidney Calculi OR Upper Ureter stone* OR urolithiasis OR kidney stone*) AND (retrograde intrarenal surgery OR RIRS OR flexible ureteroscopy OR flexible Ureterscopy Surgeries) AND (minimally invasive percutaneous nephrolithotomy OR ultra-mini percutaneous nephrolithotomy OR miniaturized percutaneous nephrolithotomy OR miniaturized PCNL)).

To avoid omissions, we manually searched the English references of the included articles.

The inclusion criteria were define using the PICOS approach : P(patients): All adult patients diagnosed with upper urinary tract stones (>1 cm); I(intervention): patients who underwent mPCNL lithotripsy; C (comparator): patients who underwent RIRS lithotripsy; O (outcome): at least one of the following outcomes; SFR, operation duration, hospitalization time, pain visual analogue score, blood transfusion, hemoglobin drop, postoperative complications and hospitalization expense; S(study type): Only RCTs in English language were included. The exclusion criteria were: I: Non-comparative or non-randomized studies; III: Editorial comments, meeting abstracts, case reports, or reviews; III: Tract sizes of mPCNL<20 F or>11 F; IV: Other studies that did not meet the inclusion criteria.

### Result parameters and data collection

Data extraction was independently performed by two reviewers as follows: I: General information: first author name, year of publication and country; II: Population characteristics: number of patients, age, body mass index (BMI), stone size, nephroscope size, lithotripsy; III: Perioperative outcomes: operative time, hemoglobin drop, transfusion rates, length of hospital stay, stone-free rate; IV: Overall complications rate, minor complications (defined as Clavien grade 1–2), major complications (defined as Clavien grade ≥ 3); and V: Pain visual analogue score, hospitalization expense. Any differences were resolved by consensus or by consultations with the third reviewer.

### Quality assessment and statistical analysis

The qualities of all included RCTs were assessed using the Cochrane Collaboration’s tool for randomized trials(ROB 2) [23], including randomization process, deviations from intended interventions, missing outcome data, measurement of the outcome, selection of the reported result, and overall bias. Meta-analysis was conducted using the Stata 15.1 software (StataSE, USA). Risk ratio (RR) was used for dichotomous variables, whereas continuous variables were pooled as weighted mean difference (WMD). Statistical heterogeneity was evaluated using *I*^2^ statistics; When *I*^2^ ≥ 50% (p ≤ 0.1), it indicated significant heterogeneity, and a random effects model was employed; when *I*^2^ < 50%, a fixed effects model was employed (p > 0.1); p ≤ 0.05 was the threshold for statistical significance [24]. Subgroup and sensitivity analyses were performed when necessary to explore the sources and sizes of heterogeneity among studies. Publication bias was screened by using the funnel plot.

## Results

### Baseline characteristics

**Fig. 1:**
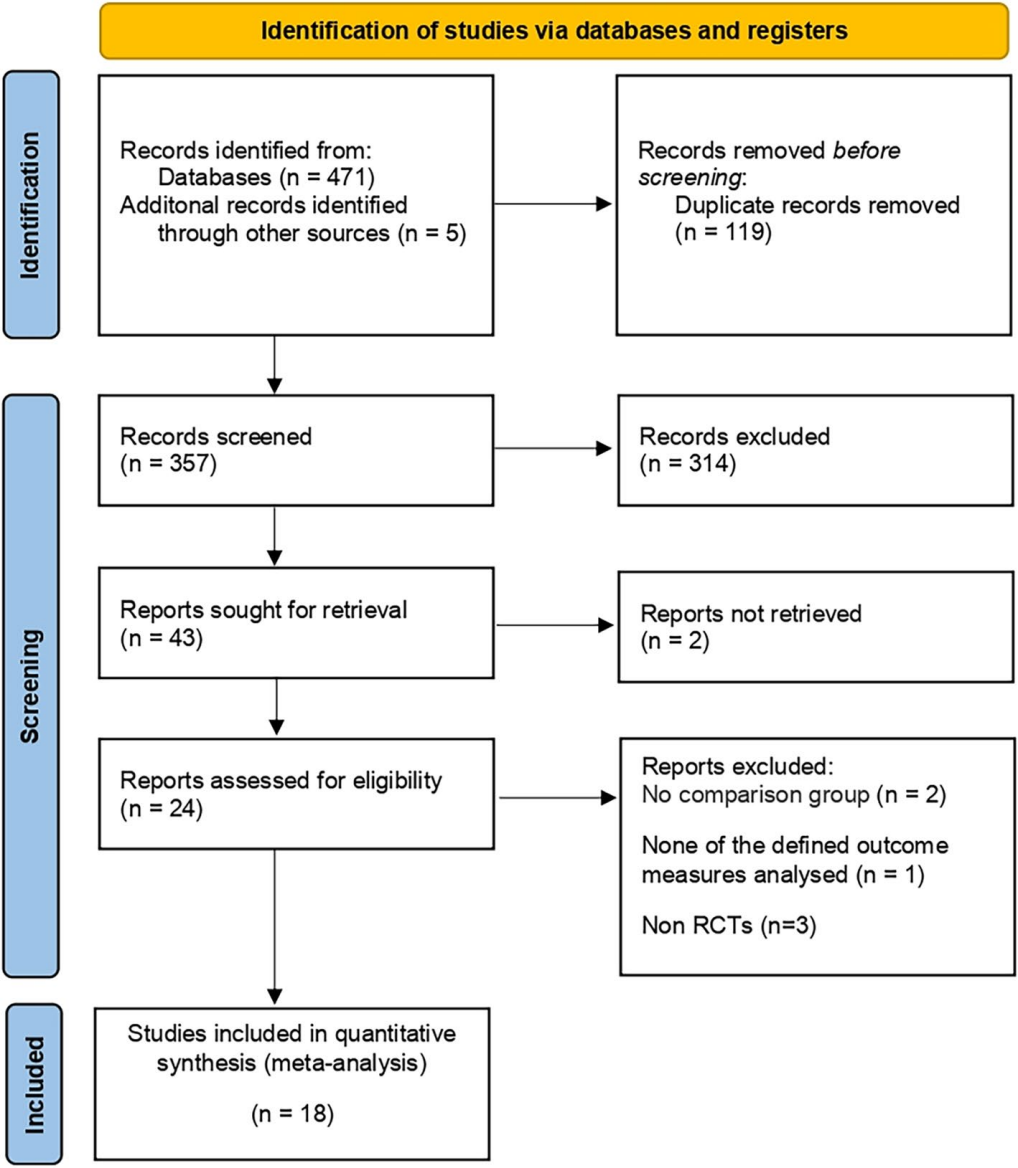
PRISMA flow diagram for the systematic review

**Fig. 2:**
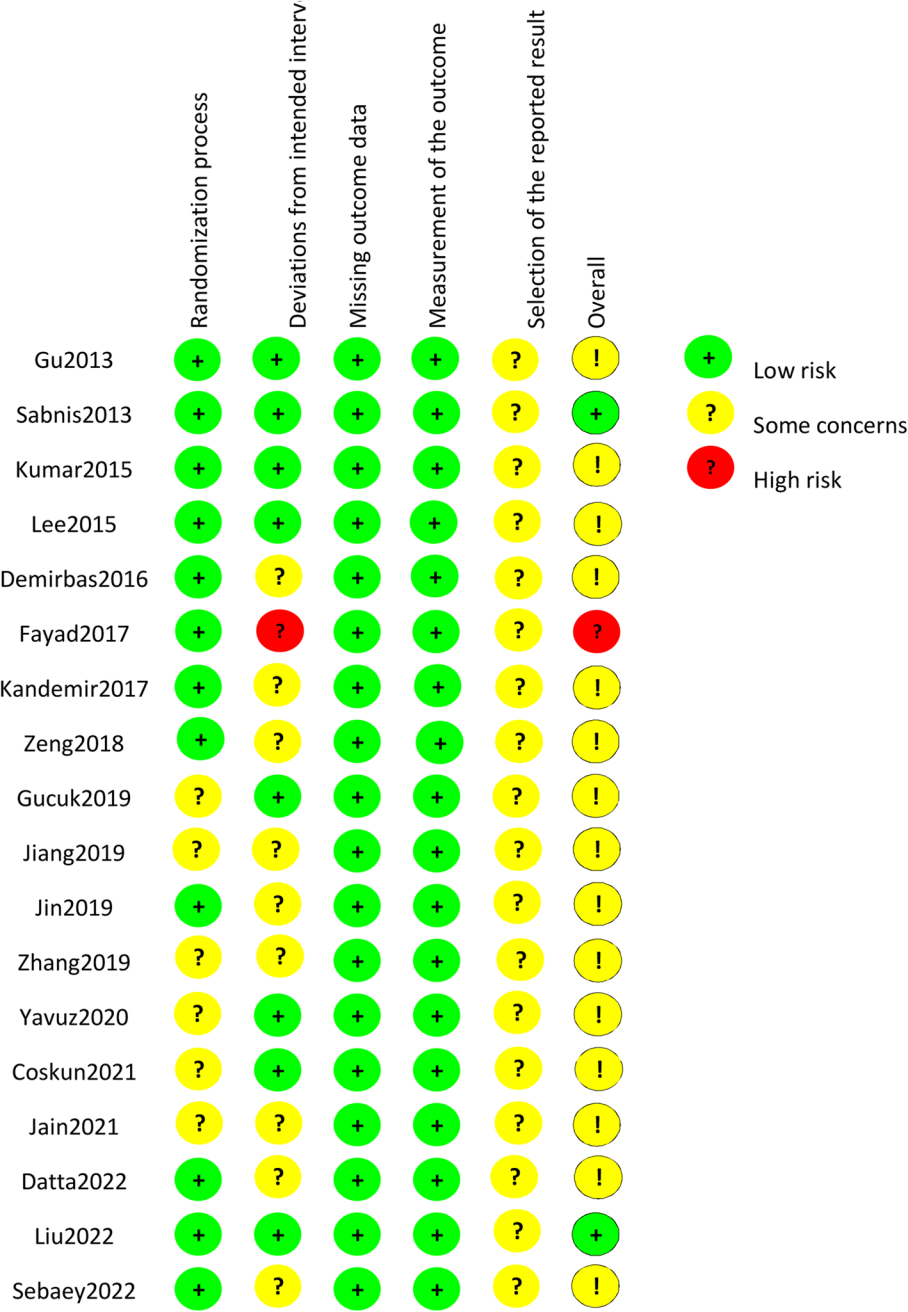
Risk of bias assessment

According to the literature screening process in Fig. 1, 18 [17–21, 25–37] qualified RCTs were included in this Meta-analysis Table 1; Fig. 2 summarizes the risk bias in the included studies. These trials included 887 mini-PCNL cases (52%) and 846 RIRS cases (48%). Table 2 summarizes the baseline characteristics and the associated preoperative variables (sample size, age, BMI, stone size, and lithotripsy) of the included patients. Outcome parameters or all included studies and the results after Meta-analysis are summarized in Tables 3 and 4.

**Table 1:** Summary of RCTs studies included in Meta-analysis

Study	Country	Study period	Study design	Sample size		mPCNL category	Definition of stone free rate	LE
				MPCNL	RIRS			
Gu 2013 [25]	2010.9-2011.11	RCT	30	29	Mini-PCNL	Fragments < 4 mm at 3 mo on NCCT	Fragments＜4 mm at 3 mo on NCCT	2a
Sabnis 2013 [26]	India	2011.2-2012.8	RCT	35	35	Micro-PCNL	Fragments < 4 mm at 3 mo on NCCT	2a
Kumar 2015 [27]	India	2012.1-2013.5	RCT	41	43	Mini-PCNL	Fragments < 4 mm at 3 mo on NCCT	2a
Lee 2015 [28]	Korea	2014.6-2015.2	RCT	35	33	Mini-PCNL	Fragments < 2 mm at 3 mo on NCCT	2a
Demirbas 2016 [29]	Turkey	2015.3-2015.9	RCT	30	43	Ultra-mini PCNL	Fragments < 3 mm at 1 mo on NCCT	2a
Fayad 2017 [30]	Egypt	2012.7-2015.12	RCT	60	60	Mini-PCNL	Fragments < 3 mm at 3 mo on NCCT	2a
Kandemir 2017 [31]	Turkey	2013.3-2015.12	RCT	30	30	Micro-PCNL	Fragments < 4 mm at 3 mo on NCCT	2a
Zeng 2018 [32]	China	2015.8-2017.7	RCT	80	80	Super-Mini PCNL	NA	2a
Gucuk 2019 [33]	Turkey	2016.4-2017.5	RCT	30	30	Mini-PCNL	No fragments at 3 mo on low dose NCCT	2a
Jiang 2019 [17]	China	2013.1-2017.3	RCT	57	56	Micro-PCNL	Fragments < 3 mm at 3 mo on NCCT	2a
Jin 2019 [18]	China	2017.5-2019.7	RCT	110	110	Mini-PCNL	Fragments < 3 mm at 3 mo on NCCT	2a
Zhang 2019 [19]	China	2015.3-2017.3	RCT	60	60	Ultra-mini PCNL	Fragments < 3 mm at 3 mo on NCCT	2a
Yavuz 2020 [34]	Turkey	2017.1-2017.12	RCT	33	34	Ultra-mini PCNL + Micro-PCNL + Mini-PCNL	Fragments < 3 mm at 3 mo on NCCT	2a
Coskun 2021 [35]	Turkey	2016.6-2016.12	RCT	25	25	Mini-PCNL	Fragments < 3 mm at 3 mo on NCCT	2a
Jain 2021 [36]	India	2016.12-2018.10	RCT	40	40	Mini-PCNL	Fragments < 4 mm at 1 mo on NCCT	2a
Datta 2022 [20]	England	2015.5-2016.12	RCT	98	46	Ultra-mini PCNL	Fragments < 2 mm at 1 mo on low dose NCCT	2a
Liu 2022 [21]	China	2018.7-2020.7	RCT	58	57	Mini-PCNL	Fragments < 3 mm at 3 mo on NCCT	2a
Sebaey 2022 [37]	Egypt	2017.9-2019.9	RCT	35	35	Mini-PCNL	Fragments < 4 mm on NCCT	2a

*LE *Eevel of evidece, *mPCNL *minimally invasive percutaneous nephrolithotomy, *RIRS R*etrograde intrarenal surgery; NA not available

**Table 2:** Characters of patients and calculus

Study	Treatments	Age(year)	BMI(kg/m^2^)	Male/Female	Stone size(mm)	Lithotripsy	Dilator	Access sheath size, Fr	Nephroscope size
Gu 2013 [25]	mPCNL	42.5 ± 10.1	NA	NA	17.27(15–25)	Laser	FD	12-18 F	8.5 F/9.8 F
	RIRS	44.22 ± 13	NA	NA	16.23(15–25)	Laser	UAS	NA	8.5 F/9.8 F
Sabnis 2013 [26]	mPCNL	38.6 ± 14.6	23.9 ± 4.9	22/13	11 ± 2.3	Laser	NA	NA	NA
	RIRS	43.7 ± 12.1	24.9 ± 4.3	24/11	10.4 ± 2.5	Laser	FD	12 F	7.5 F
Kumar 2015 [27]	mPCNL	33.7 ± 1.6	23.5 ± 1.2	20/21	13.3 ± 1.3	Pneumatic	NA	18 F	15 F
	RIRS	33.4 ± 1.4	23.6 ± 1.1	20/23	13.1 ± 1.1	Laser	UAS	12 F	8/9.8 F
Lee 2015 [28]	mPCNL	59.3 ± 13.3	26.3 ± 3.9	28/7	39.1 ± 30.7	Laser	Balloon dilator	18 F	15 F
	RIRS	55.8 ± 11.2	25.6 ± 5.1	28/5	28.9 ± 17.5	Laser	UAS	12/14/16F	7.5 F
Demirbas 2016 [29]	mPCNL	47.7 ± 14.6	NA	21/9	185.9 ± 88.3	Laser	Amplatz dilators	14 F	6/7.5 F
	RIRS	48.7 ± 16.9	NA	20/23	181.7 ± 114.18^a^	Laser	UAS	11.5 F	7.5 F
Fayad 2017 [30]	mPCNL	37.2 ± 9.2	NA	38/22	14.7 ± 3	Laser	Alkan dilators	16 F	10 F
	RIRS	37.7 ± 9.8	NA	34/26	14.1 ± 3	Laser	UAS	12/14F	7.5 F
Kandemir 2017 [31]	mPCNL	49.7(1–78)	NA	16/14	10.6(5–15)	Laser	NA	16 F	NA
	RIRS	51.8(21–81)	NA	19/11	11.5(7–15)	Laser	UAS	NA	NA
Zeng 2018 [32]	mPCNL	49.4 ± 12.8	23.26 ± 3.41	36/17	15 ± 2.9	Laser	FD	14 F	NA
	RIRS	47.1 ± 13.9	23.63 ± 3.83	39/14	14.3 ± 3.4	Laser	UAS	12/14F	NA
Gucuk 2019 [33]	mPCNL	46.1 ± 17.5	26.4 ± 3.3	21/9	275.5 ± 75.1	Laser	One step dilator	16.5 F	12 F
	RIRS	46.6 ± 13.5	27.2 ± 3.7	23/7	259.1 ± 65.2^b^	Laser	UAS	9.5/11.5 F	7.5 F
Jiang 2019 [17]	mPCNL	43.4 ± 11.6	23.9 ± 4.5	39/19	16.1 ± 3.0	Laser	NA	16 F	NA
	RIRS	45.4 ± 11.2	24.1 ± 4.3	42/16	15.2 ± 3.2	Laser	UAS	14 F	7.5 F
Jin 2019 [18]	mPCNL	53.2 ± 13.7	24.8 ± 3.8	79/31	14.9 ± 3.9	Laser	FD	16 F	NA
	RIRS	51.4 ± 11.9	25.3 ± 4.2	72/38	16.4 ± 3.3	Laser	UAS	12/14F	8/9.8 F
Zhang 2019 [19]	mPCNL	48.9 ± 11.1	24.31 ± 3.01	37/23	15.48 ± 2.45	Laser	UMP	13 F	4.5 F
	RIRS	50 ± 11.9	24.33 ± 3.14	34/26	14.63 ± 2.67	Laser	UAS	12/14F	NA
Yavuz 2020 [34]	mPCNL	42.3 ± 12.7	24.6 ± 3.7	18/16	415 ± 82	Laser	NA	12 F	4.5 F
	RIRS	48.1 ± 13.1	25.4 ± 2.8	20/13	401 ± 85^a^	Laser	UAS	12 F	6/7.5 F
Coskun 2021 [35]	mPCNL	44 ± 14	NA	15/10	15.7 ± 2.5	Pneumatic	Amplatz dilators	NA	NA
	RIRS	48 ± 13.9	NA	13/12	13.6 ± 2.2	Laser	UAS	NA	5.5 F
Jain 2021 [36]	mPCNL	35.6	23	25/15	12.35	Laser/Pneumatic	FD	16.5/17.5	16 F
	RIRS	40.45	25.09	32/8	12.9	Laser	URF	11 F	NA
Datta 2022 [20]	mPCNL	39.08	23.59	59/39	16.31	Laser	NA	13 F	NA
	RIRS	40.54	24.27	29/17	16.02	Laser	NA	NA	NA
Liu 2022 [21]	mPCNL	47.59 ± 10.9	32.62 ± 1.94	39/19	585.39 ± 131.06^a^	Laser	FD	18 F	12 F
	RIRS	49.19 ± 13.3	31.19 ± 2.04	40/17	548.64 ± 123.55^a^	Laser	UAS	12/14F	NA
Sebaey 2022 [37]	mPCNL	36.11 ± 11.91	41.76 ± 9.34	25/10	20.43 ± 2.2	Laser	Teflon dilators	14 F	12 F
	RIRS	34.0 ± 10.69	42.21 ± 10.22	17/18	20.5 ± 2.1	Laser	USA	12/14F	7.5 F

^a^Stone size is presented in terms of length (mm) unless indicated otherwise: mm^3^; ^b^ Stone size is presented in terms of length (mm) unless indicated otherwise: mm^2^ ; *mPCNL *minimally invasive percutaneous nephrolithotomy, *RIRS *Retrograde intrarenal surgery, *UAS *Ureteral access sheath placement, *FD *Fascial dilators, *NA *Not available;

**Table 3:** Outcome parameters of MPCNL and RIRS

Study	Treatments	SFR(%)	Hospitalization time(day)	Operation	Postoperative	Hb drop(g/dl)	Transfusion,n(%)	cost	Complications(%)			
				duration(min)	pain score				Overall n(%)	Grade I n(%)	Grade II n(%)	Grade III n(%)
Gu 2013 [25]	mPCNL	100	4.6 ± 1.8	50–135(96.2)	-	-	-	-	-	-	-	-
	RIRS	89.7	1.9 ± 1.3	45–100(66.8)	-	-	-	-	-	-	-	-
Sabnis 2013 [26]	mPCNL	97.1	2.4 ± 0.9	51.6 ± 18.5	1.9 ± 1.2	0.96 ± 0.41	-	-	9(25.7)	8(22.9)	1(2.8)	0
	RIRS	94.3	2.1 ± 0.75	47.1 ± 17.5	1.6 ± 0.8	0.56 ± 0.31	-	-	4(11.4)	4(11.4)	0	0
Kumar 2015 [27]	mPCNL	95.1	3.1	61.1 ± 1.3	-	-	12.9	-	10(24.9)	8(20)	2(4.9)	-
	RIRS	86.1	1.3	47.5 ± 1.1	-	-	0	-	4(9.4)	2(4.7)	2(4.7)	-
Lee 2015 [28]	mPCNL	85.7	1.6 ± 1.1	76.1 ± 70.6	4.2 ± 2.6	0.69 ± 0.98	-	-	15(42.9)	11(31.4)	4(11.4)	0
	RIRS	97	1.5 ± 0.9	99.6 ± 60.8	5.7 ± 3.0	0.38 ± 0.97	-	-	22(62.9)	19(54.3)	3(9.1)	0
Demirbas 2016 [29]	mPCNL	80	2.46 ± 3.02	54.53 ± 23.09	4.73 ± 1.25	-	-	665	7(23.4)	2(6.7)^a^		5(16.7)
	RIRS	74.4	1.37 ± 1.48	59.41 ± 15.78	2.30 ± 1.12	-	-	1160	6(14)	3(7)^a^		3(7)
Fayad 2017 [30]	mPCNL	92.72		71.66 ± 10.36	-	0.28	0	-	5(8.33)	5(8.33)	-	-
	RIRS	84.31		109.66 ± 20.75	-	0.13	0	-	5(8.33)	5(8.33)	-	-
Kandemir 2017 [31]	mPCNL	93.7	54.2	59.04	-	1.06(0.1–28)	3.3	-	5(16.6)	4(13.3)	1(3.3)	0
	RIRS	86.7	19	51.05	-	0.75(0.1–21)	0	-	6(19.9)	4(13.3)	2(6.6)	0
Zeng 2018 [32]	mPCNL	93.8	2.5 ± 1.1	58.6 ± 21.6	2.7 ± 1.7	1.02 ± 0.89	0	-	6(7)	9(11.7)	2(2.6)	-
	RIRS	82.5	2.5 ± 1.1	52.3 ± 22.4	2.0 ± 1.5	0.43 ± 0.88	0	-	6(7)	12(14.5)	2(2.6)	-
Gucuk 2019 [33]	mPCNL	86.7	2.1 ± 2.03	98.3 ± 18.8	3.1 ± 1.4	-	3.3	-	12(40)	9(30)	3(10)	0
	RIRS	83.3	1.6 ± 1.34	109.0 ± 33.8	3.0 ± 1.4	-	0	-	9(30)	6(20)	1(3.3)	2(6.6)
Jiang 2019 [17]	mPCNL	94.7	3.2 ± 0.5	54.0 ± 8.2	-	3.0 ± 2.3	**-**	-	4(6.9)	3(5.2)	1(1.7)	0
	RIRS	92.9	3.2 ± 0.6	60.3 ± 8.5	-	2.3 ± 1.5	**-**	-	6(5.2)	0	4(3.4)	2(1.7)
Jin 2019 [18]	mPCNL	99.1	5.59 ± 0.82	79.6 ± 14.86	3.42 ± 1.24	1.14 ± 0.76	**-**	-	12(11)	0	12(11)	0
	RIRS	97.3	3.15 ± 0.72	87.2 ± 13.34	1.62 ± 0.86	0.98 ± 0.68	**-**	-	6(5.5)	0	6(5.5)	0
Zhang 2019 [19]	mPCNL	98	5.3 ± 1.2	68.58 ± 15.82	-	0.89	1.6	4085.51 ± 416.69	10(16.7)	2(3.3)	5(8.3)	3(5)
	RIRS	92	3.2 ± 0.5	93.35 ± 21.64	-	0.44	0	4657.28 ± 679.28	6(10)	2(3.3)	3(5)	1(1.7)
Yavuz 2020 [34]	mPCNL	94.1	2 (1–14)	61.6 ± 18.5	-	-	-	632 ± 314	3(8.8)	-	3(8.8)	0
	RIRS	76	1 (0.5-3)	60.7 ± 13	-	-	-	1250 ± 505	2(6)	-	2(6)	0
Coskun 2021 [35]	mPCNL		4.6 ± 3,5	71.7 ± 24.4	-	-	8	-	-	7(28)	9(36)	9(36)
	RIRS		1.2 ± 0.59	72.8 ± 24.2	-	-	0	-	-	20(78)	3(12)	0
Jain 2021 [36]	mPCNL	92.5	2.85	51.58	-	0.88	-	-	9(22.5)	2(5)	3(7.5)	4(10)
	RIRS	87.5	2.45	69.75	-	0.42	-	-	16(40)	4(10)	7(17.5)	5(12.5)
Datta 2022 [20]	mPCNL	100	39.21	41.17	-	0.46	-	45.61	10(10.3)	6(6.2)	4(4.1)	0
	RIRS	73	39.08	73.58	-	0.31	-	423.02	16(35)	16(35)	0	0
Liu 2022 [21]	mPCNL	86.2	3.5 ± 1.58	48.2 ± 24.25	4.6 ± 1.34	0.98 ± 0.55	5.2	-	13(22.4)	13(22.4)	-	-
	RIRS	61.4	2.5 ± 1.24	43.5 ± 17.23	3.9 ± 1.07	0.76 ± 0.58	0	-	4(7)	4(7)	-	-
Sebaey 2022 [37]	mPCNL	88.6	1.41 ± 0.46	59.71 ± 19.44	-	1.27 ± 0.1	2.9	-	3(8.6)	3(8.6)	-	-
	RIRS	82.9	1.29 ± 0.44	80.43 ± 14.79	-	1.29 ± 0.1	0	-	1(2.9)	1(2.9)	-	-

^a^overall number of Grade I and Grade II; m*PCNL *minimally invasive percutaneous nephrolithotomy, *RIRS *Retrograde intrarenal surgery, *Hb *Hemoglobin, *SFR *Stone free rate

**Table 4:** Results of meta-analysis comparing mPCNL and RIRS

Outcomes	No.of studies	SamPle size		Heterogeneity(Total)				W(S)MD/RR(95%CI)	P value(Total)
		mPCNL	RIRS	chi^2^	df	I^2%^	*P* value		
Overall SFR	17	862	821	42.3	16	62.1	0.01	1.08(1.03–1.14)	*P* = 0.002
SFR(stone 1–2 cm)	11	571	573	28.34	10	64.7	0.002	1.09(1.03–1.15)	*P* = 0.003
SFR(stone 2–3 cm)	3	123	122	4.78	2	58.2	0.092	1.19(1.05–1.36)	*P* = 0.007
Operation time	18	887	846	836.6	17	98.00%	0	-6.82(-15.45, 1.81)	*P* = 0.122
Operation time(2013–2019)	8	341	353	322.5	7	97.80%	0	-0.65(-16.56, 15.26)	*p* = 0.936
Operation time(2019–2022)	10	546	493	62.9	9	85.70%	0	-10.85(-16.76,-4.94)	*p*<0.001
Transfusion	9	419	420	0.78	6	0	0.993	5.04(1.62–15.65)	*P* = 0.005
Hospitalization time	17	827	786	810.5	16	98	0	1.01(0.53,1.5)	*P*<0.001
Pain visual analogue score	7	378	388	81.65	6	92.7	0	0.75(0.04–1.46)	*P* = 0.037
Hb drop	11	723	667	56.4	10	82.3	0	0.27(0.14–0.41)	*P*<0.001
Complication ratio	16	832	792	38.29	15	60.8	0.001	1.17(0.82–1.68)	*P* = 0.39
Clavien-Dindo(I-II)	17	857	817	40.47	16	60.5	0.001	1.03(0.74–1.42)	*P* = 0.88
Clavien-Dindo(III)	12	583	542	8.25	7	15.2	0.311	1.89(1.014–3.53)	*P *= 0.045
Cost	4	221	183	5.18	3	42	0.159	-0.97(-1.19, -0.76)	*P*<0.001

*CI *Confidence interval, *WMD *Weighted mean difference, *SMD *Standardized mean difference, *mPCNL *Minimally invasive percutaneous nephrolithotomy, *RIRS *Retrograde intrarenal surgery, *RR *Risk ratio, *SFR *Stone free rate;

### Outcome analysis

#### Overall stone free rate

**Fig. 3:**
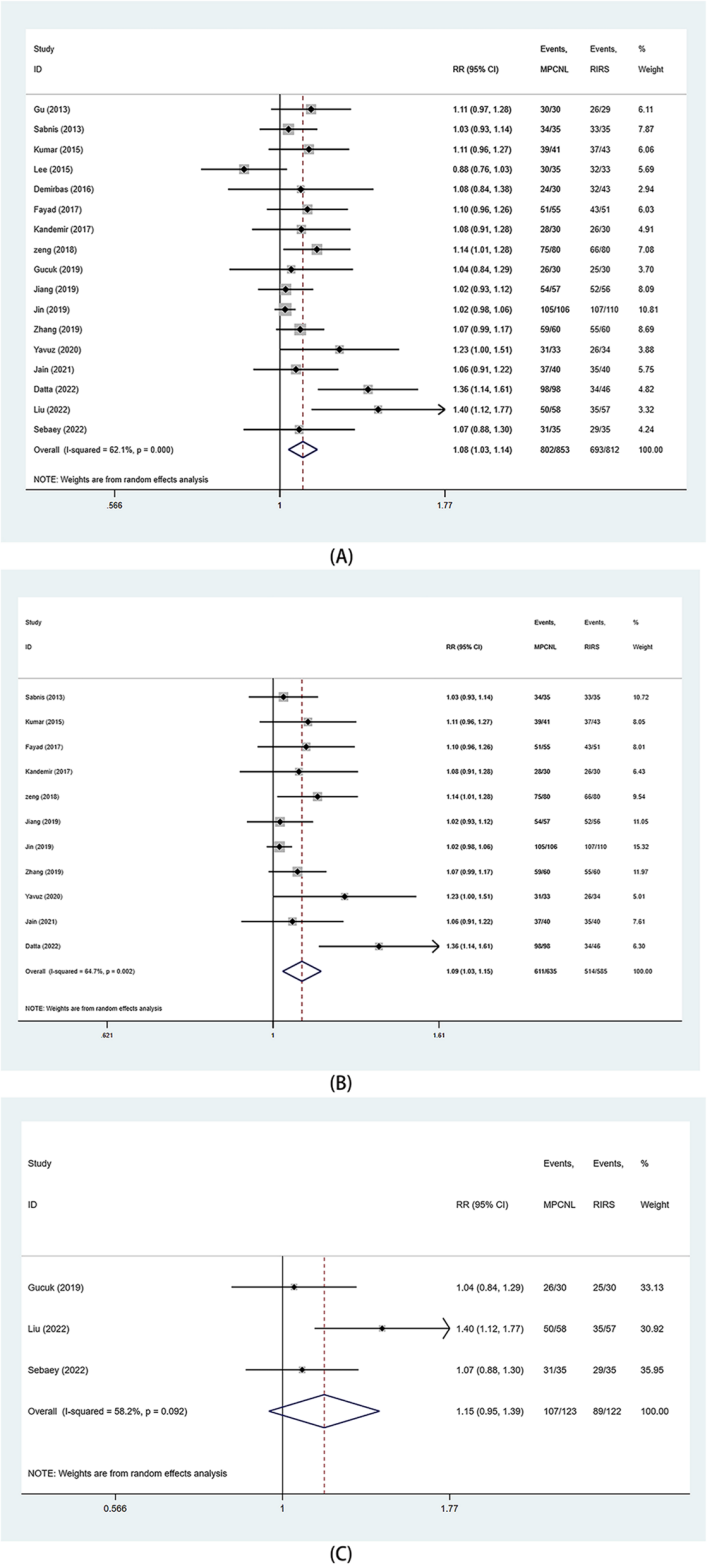
Forest plots of SFR. **A** overall SFR; **B** SFR (stone 1-2 cm); **C**. SFR (stone>2 cm)

Seventeen studies reported on SFR. Due to heterogeneity (> 50%), a random effects model was employed for analysis. The SFR was found to be significantly higher in the mPCNL group, relative to the RIRS group (RR: 1.08, 95%CI 1.03, 1.14 *p* = 0.002) (Fig. 3A**)**. Moreover, significant outcomes were obtained in subgroups with stone sizes of 1–2 cm (RR: 1.09, 95%CI 1.03, 1.15 *p* = 0.003) (Fig. 3B**)** and 2–3 cm (RR: 1.19, 95%CI 1.05, 1.36 *p* = 0.007) (Fig. 3C).

#### Operation duration

**Fig. 4:**
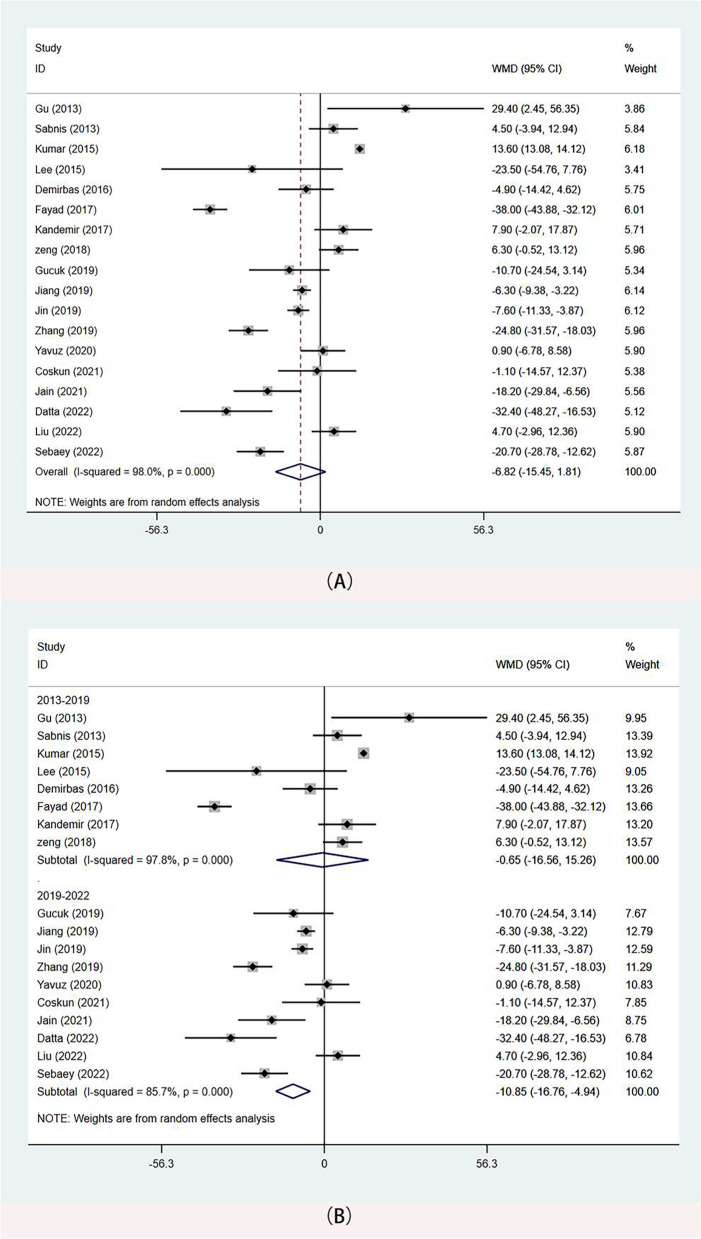
Forest plots of operative time. **A** operative time(Overall); **B** operative time (2019–2022)

The operative time was reported in all included studies, and analyses were conducted using the random-effects model. The meta-analysis did not reveal any differences between the two surgical methods (WMD: -6.82 min, 95%CI -15.45, 1.81 *p* = 0.122) (Fig. 4A**).** However, subgroup analysis according to publication time showed that for studies published after 2019, procedure time was better in the mPCNL group than in the RIRS group (WMD: -10.85 min, 95%CI -16.76, -4.94 *p*<0.001) (Fig. 4B).

#### Length of hospital stay

**Fig. 5:**
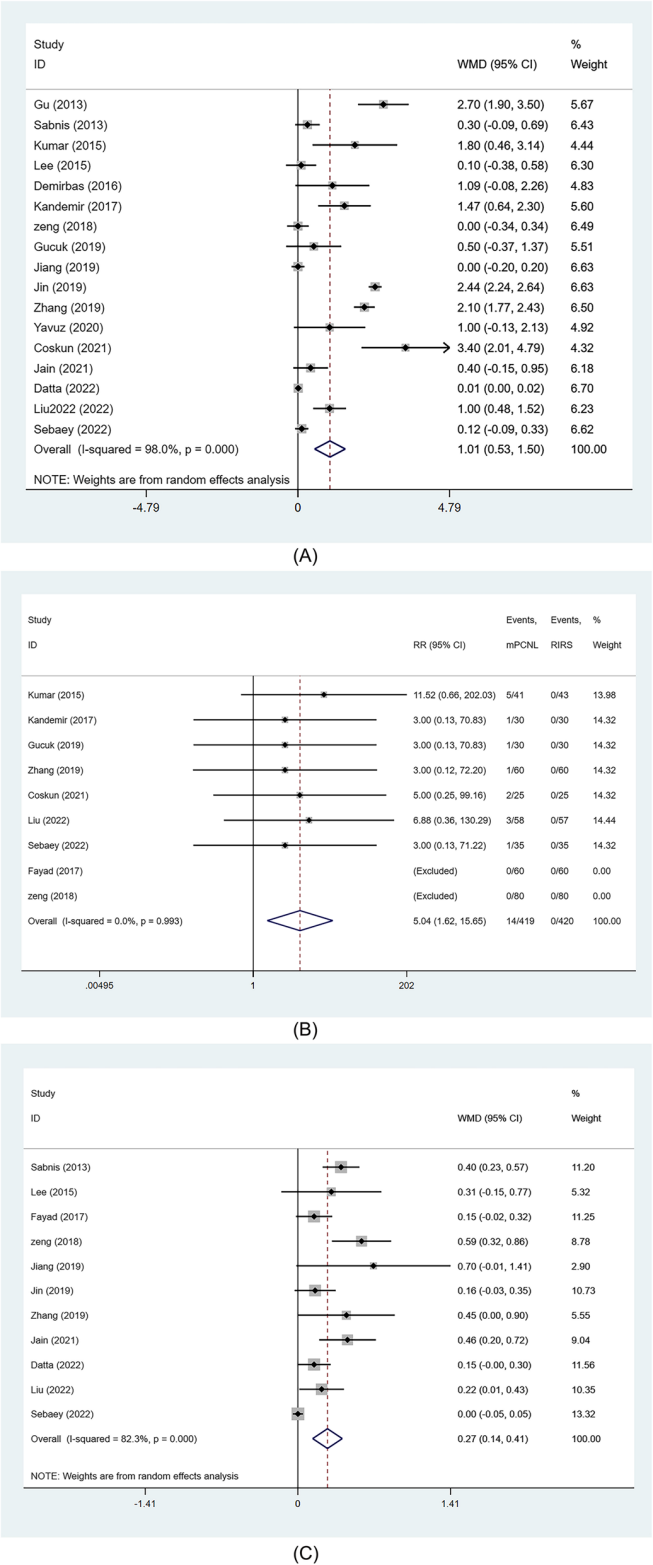
Forest plots of Los, transfusion rate and hemoglobin drop. **A** Length of stay; **B** transfusion rate; **C.** hemoglobin drop

Data on length of hospital stay were extracted from 17 studies. The random-effects model showed that the length of hospital stay was significantly longer in the mPCNL group than in the RIRS group (WMD: 1.01 day, 95%CI 0.53, 1.5 *p*<0.001) (Fig. 5A**).**

#### Transfusion rate

Nine studies reported on the occurrence of transfusion events. The meta-analysis using a fixed-effects model showed that the probability of blood transfusion was significantly higher in the mPCNL group relative to the RIRS group (RR: 5.04, 95%CI 1.62, 15.65, *p* = 0.005) (Fig. 5B**).**

#### Hemoglobin drop

Data were obtained from 11 studies. The random-effects model revealed a greater decrease in hemoglobin levels in the mPCNL group than in the RIRS group (WMD 0.27 g/dl, 95%CI 0.14–0.41, *p*<0.001) (Fig. 5C).

#### Pain visual analogue score

**Fig. 6:**
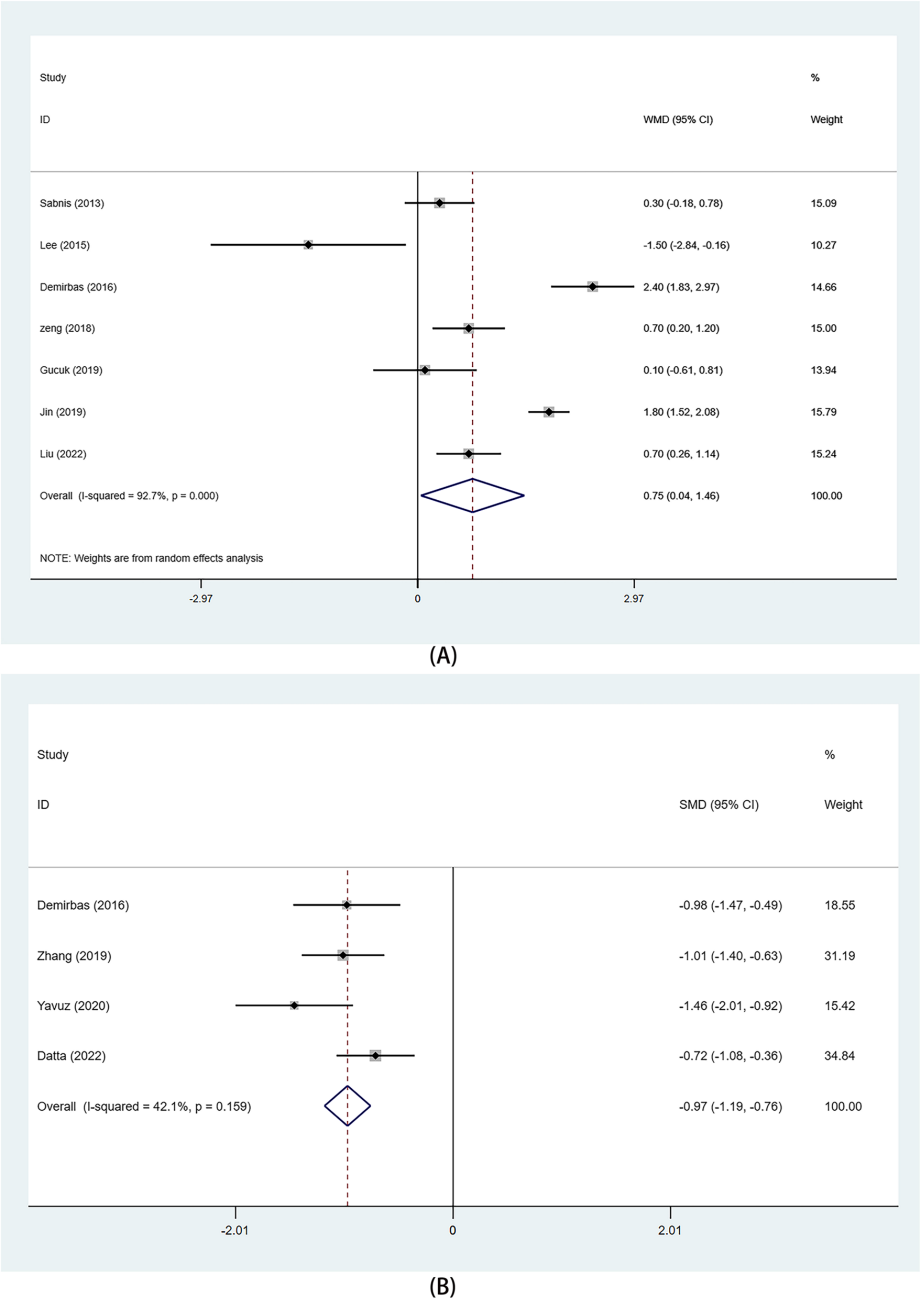
Forest plots of pain visual analogue score and Hospitalization expense. **A** pain visual analogue score; **B** Hospitalization expense

Data were derived from 7 studies. The random-effects model showed that patients in the mPCNL group had higher scores than those in the RIRS group (WMD: 0.75, 95%CI 0.04, 1.46, *p* = 0.037)(Fig. 6A).

#### Hospitalization expense

Among the studies, only four reported hospital costs. In the fixed-effects analysis, patient costs were generally lower in the mPCNL group than in the RIRS group (SMD: -0.97, 95%CI (-1.19, -0.76), *p*<0.001)(Fig. 6B).

#### Postoperative Complications

**Fig. 7:**
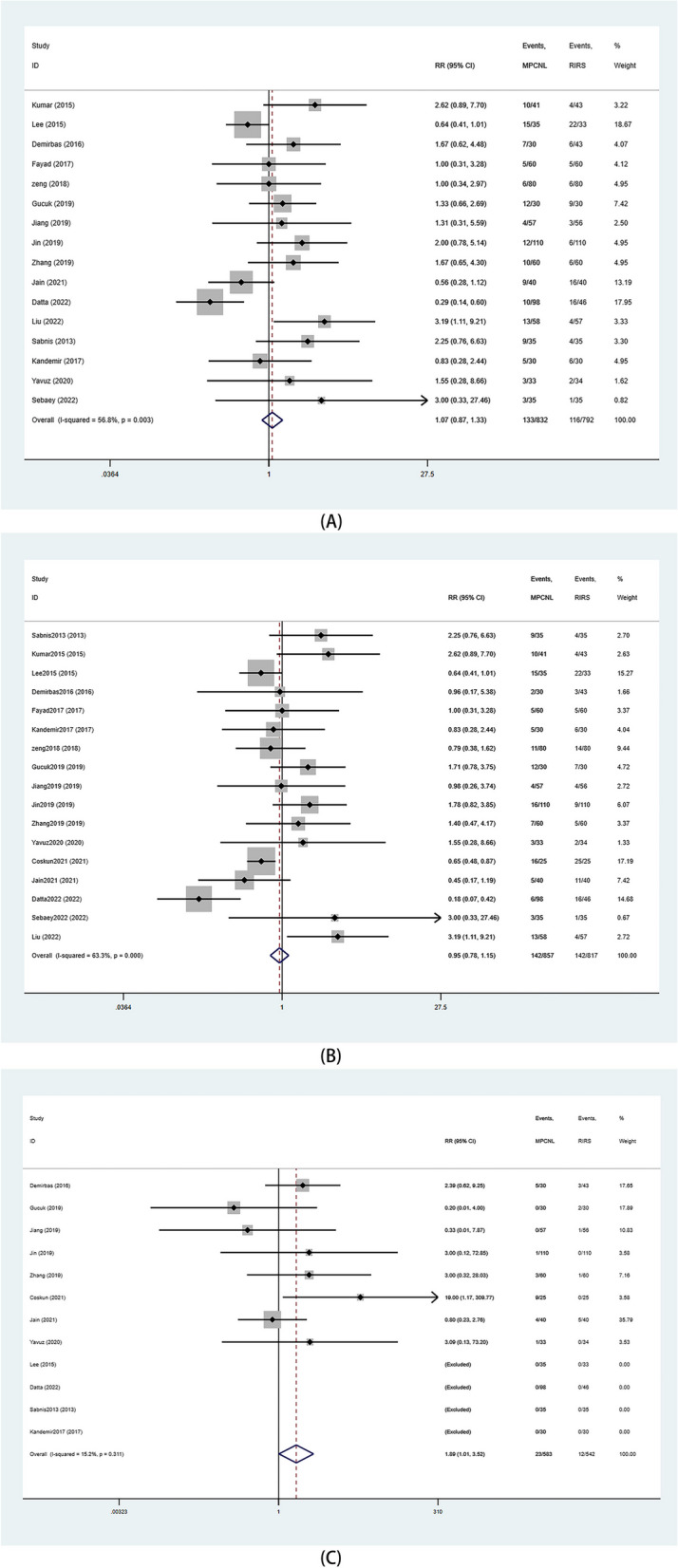
Forest plots of complication. **A** overall complication; **B** minor complication; **C** major complication

Postoperative complications were classified as follows: overall complications rate, minor complications (defined as Clavien grade 1–2), major complications (defined as Clavien grade ≥ 3) using the Clavien-Dindo classification system. In the random-effects model, we did not find differences in overall complications between the two surgical procedures (RR: 1.07 95%CI 0.87, 1.33 *p* = 0.51) (Fig. 7A). Data from 17 studies also showed no difference in the rate of minor complications between the groups (RR: 0.95 95%CI 0.78, 1.15 *p* = 0.58) (Fig. 7B). However, results of the fixed-effects model showed that the rate of severe complications was higher for mPCNL than for RIRS (RR: 1.89 95%CI 1.01–3.53 *P* = 0.045) (Fig. 7C).

#### Heterogeneity

The majority of outcomes showed moderate to high heterogeneity, with only transfusion rates, major complications, and hospitalization costs having low heterogeneity outcomes. However, the reported low or moderate heterogeneity may be misleading because I^2^ is highly biased in a small number of studies [38]. Certain outcome measures (OT, LOS, and Hb drop) exhibit substantial heterogeneity. We endeavored to mitigate confounding factors such as country, publication year, and mPCNL type through meta-regression analysis. No discernible sources of heterogeneity were identified (p > 0.05) (Supplementary File 1). Furthermore, we conducted subgroup analyses for each outcome measure based on mPCNL type. The results indicate statistical significance only in terms of operative time, with no discernible differences observed in the remaining metrics (Supplementary File 2).

#### Publication bias

**Fig. 8:**
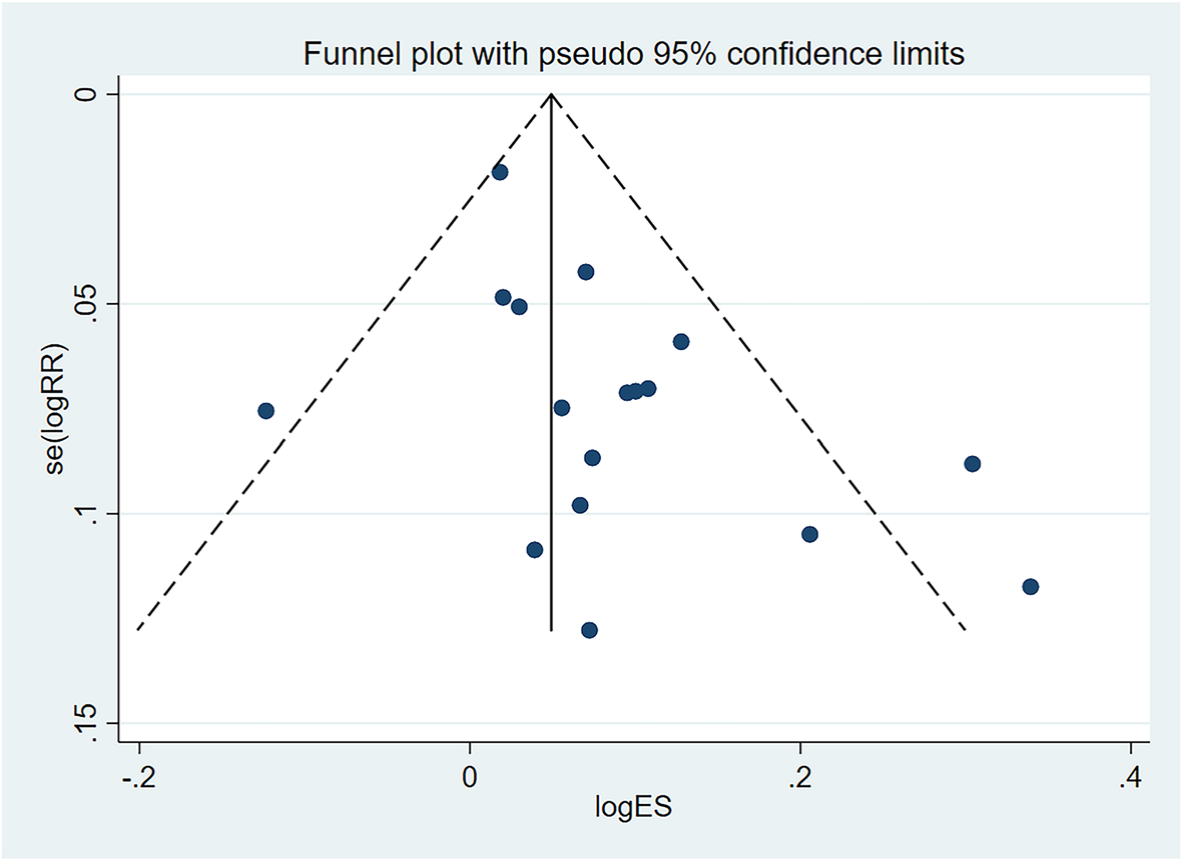
Funnel plot of operative time for publication bias

Analysis of the funnel plot revealed no significant asymmetry, indicating that there was no significant publication bias in our results (Fig. 8).

## Discussion

This is a meta-analysis incorporating the latest research. Our study reveals that, compared to RIRS, mPCNL exhibits advantages such as shorter operation time and higher stone free rate. However, in terms of hospital stay time, transfusion rate, pain visual analogue score, and major complications, mPCNL performance falls short of that of RIRS. These findings warrant further discussion.

In recent years, minimally invasive surgery has increasingly been applied in the treatment of upper urinary tract calculi. According to the guidelines of European Association of Urology, both pcnl and RIRS are recommended for the treatment of upper urinary tract stones with diameter < 2 cm, and PCNL is the first-line treatment for upper urinary tract stones with diameter > 2 cm [39]. With the rapid development of minimally invasive technology and endoscopic instruments, traditional PCNL has been constructed from Mini-PCNL, Ultra-mini PCNL, Super-mini PCNL, Micro-pcnl and other minimally invasive PCNL procedures. Such improvements have substantially decreased the access and size of the puncture sheath of the previous 20 F [40]. Compared to conventional PCNL, miniaturized PCNL (mPCNL) has demonstrated a distinct advantage in reducing postoperative complications [41–43]. Over the years, traditional RIRS has also evolved to become today’s dual-channel flexible ureteroscope, disposable flexible ureteroscope, robot-assisted flexible ureteroscope and other new flexible ureteroscope [44, 45]. In the comparison of advantages and disadvantages of mPCNL and RIRS, it is not only necessary to pay attention to the stone clearance rate, but also to the quality of life during the perioperative period. In this paper, we analyzed the most recent RCT study to comprehensively compare the advantages and disadvantages of the two surgical methods in the treatment of upper urinary calculi.

The efficacy of the two treatments is based on total stone-free rate. This study found that mPCNL had a better summary stone-free rate compared with RIRS, which was consistent with results of a previous meta-analysis [46]. However, for stones measuring 2 to 3 centimeters, there is no significant difference in the effectiveness between the two surgical procedures. In this study, subgroup analyses revealed that for stones measuring 1–2 centimeters and 2–3 centimeters, mPCNL demonstrated superiority over RIRS. This observation may be attributed to the steeper funnel-pelvic angle and longer funnel calyx, which pose challenges for RIRS in accessing the lower pole, thus limiting complete stone extraction [47]. In the study conducted by Datta et al. [20] approximately 46% of patients in the RIRS group presented with preoperative lower pole renal calyx stones, indicative of residual stones postoperatively. Ripple et al. [48] posit that patients with preoperative renal calculi larger than 1 cm, following Retrograde Intrarenal Surgery (RIRS), exhibit residual fragments in approximately 50% of cases. Furthermore, Ghan and colleagues along with WOLF et al. [49] employing rigorous CT imaging as a follow-up modality, have derived a post-RIRS average stone clearance rate of 77%. Therefore, for renal lower pole calculi that present challenges in accessibility via RIRS or are constrained by ureteral stenosis, mPCNL emerges as an impeccable alternative. In terms of operation time, there seemed to be no significant advantage or disadvantage between the two surgical methods. However, subgroup analysis reported that 10 RCTS published after 2019 showed that the operation time of mPCNL was significantly shorter compared with that of RIRS. RIRS entails a segmented surgical approach, where flexible ureteroscopy necessitates manipulation and flexion for stone fragmentation. Concurrently, mPCNL is also a multi-step procedure, involving an initial ultrasound or fluoroscopy-guided entry, placement of a guidewire within the system, removal of the initial puncture needle from the guidewire, continuous or stepwise dilation of the urethra during the procedure, resulting in urethral bleeding, and ultimately the placement of a sheath onto the urethral dilator [50]. These discrete maneuvers are time-consuming. Although mPCNL represents a relatively newer surgical modality, it is readily graspable by any urologist who has undergone PCNL training. We hypothesize that due to the accumulated experience in these procedures, contemporary practitioners exhibit greater proficiency compared to their initial application of mPCNL. The reduction in hemoglobin matched with a significant difference in blood transfusion, and the study by Kumar [27] included five patients who required blood transfusion, possibly for reasons related to the surgical technique. The magnitude of blood loss during PCNL is contingent upon the caliber of the tract [51, 52]. Employing a single-step procedure along with the use of minimally sized tracts serves to mitigate hemorrhagic tendencies and associated complications in PCNL. Notably, Desai et al. have successfully implemented the microperc technique to accomplish PCNL in a singular procedural step [53].

According to the CLAVIEN-Dindo classification, there was no significant difference in the total complications and mild complications between the two groups, but the probability of severe complications was higher in mPCNL. The principal operative-related complications associated with PCNL encompass significant hemorrhage (7.8%), renal pelvis perforation (3.4%), pleural effusion (1.8%), and transfusion (5.7%) [54]. RIRS constitutes an endoscopic procedure conducted via natural orifices, thereby minimizing trauma to the renal parenchyma and reducing intraoperative blood loss. The primary complications of RIRS involve ureteral avulsion or perforation [55]. Deployment of a ureteral access sheath may potentially result in ureteral wall injury [56]. In the study by COSKUN et al [35], the number of major complications which affected the balance were reported but no explanation was given. One possible reason could be the surgical management procedures at local hospitals. Postoperative complications of RIRS, which the authors attributed to catheter detachment, catheter displacement or calcification due to bipolar catheters, increased the risk of readmission.

RIRS typically entails the placement of stents, resulting in associated discomfort for patients and necessitating subsequent stent removal [57]. Additionally, flexible ureteroscopy is prone to wear and tear, potentially requiring significant refurbishment after 4–14 uses [58]. Disposable components like baskets can also escalate the overall procedural expenses, whereas mPCNL, characterized by lower wear rates, can yield cost-effectiveness benefits.

### Previous meta-analyses

In recent years, several studies have compared the treatment of renal stones using mPCNL and RIRS. Several studies predominantly encompassed cohort designs, potentially introducing bias in their conclusions due to the absence of randomized controlled trials [12, 59]. Moreover, despite the inclusion of RCTs, subgroup analyses based on stone size and mPCNL were not conducted due to insufficient data volume [13–15, 46].

### Strengths and limitations

Firstly, this study encompassed 18 randomized controlled trials characterized by high methodological quality and substantial sample sizes. Secondly, in contrast to previous investigations, novel findings emerged, demonstrating that mPCNL exhibited superior surgical duration and clearance rates for upper urinary tract stones measuring 2 to 3 centimeters, as compared to the RIRS group. However, mPCNL demonstrated higher levels of bleeding, transfusion rates, costs, and postoperative discomfort when compared to the RIRS group. Thirdly, we conducted relevant subgroup analyses to minimize outcome heterogeneity. Nonetheless, this study is not without limitations. Firstly, certain included studies lacked descriptions regarding blinding and randomization concealment, potentially introducing biases into the conclusions. Furthermore, variations in outcome definitions and measurement methods may contribute to outcome heterogeneity. Consequently, caution is advised when interpreting our research findings.

## Conclusions

MPCNL has a higher stone clearance rate and a shorter operation time in stones < 3 cm when both procedures are safe and effective. However, MPCNL has more hospital stay, more blood loss, more blood transfusion, more severe complications, more pain and more hospital costs than RIRS because of its invasive characteristics. Because of the high heterogeneity of some of the results, our results should be interpreted with caution, and clinicians should fully consider the advantages and disadvantages of the two surgical procedures to make the decision that is best for patients.

### Supplementary Information

**Additional file 1.**

**Additional file 2.**

## Data Availability

The data that support the findings of this study are available from the corresponding author upon reasonable request.
