# Supplementary material for: Efficacy and safety of minimally invasive percutaneous nephrolithotomy versus retrograde intrarenal surgery in the treatment of upper urinary tract stones (> 1 cm): a systematic review and meta-analysis of 18 randomized controlled trials
Source: BMC Urol. 2023 Oct 24;23:171. doi: 10.1186/s12894-023-01341-3 (PMC10598962; doi:10.1186/s12894-023-01341-3)

Meta regression of operative time


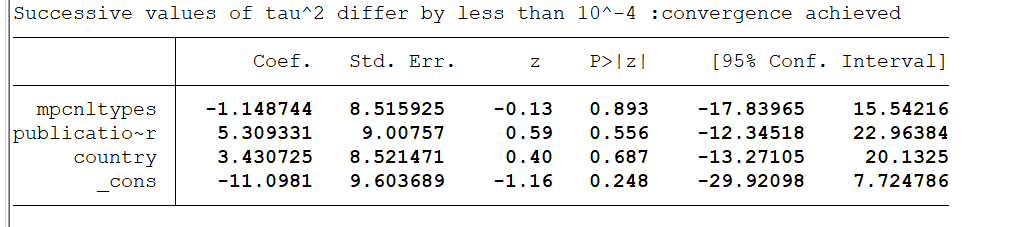


Meta regression of operation time（2013-2019）


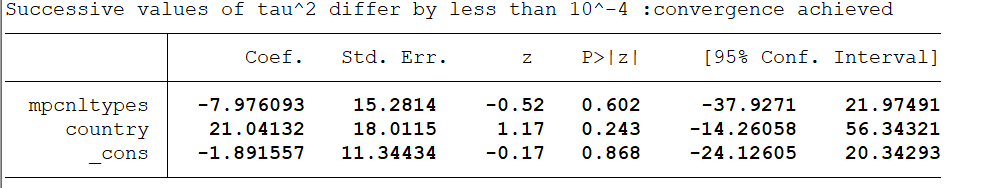


Meta regression of operation time（2019-2022）


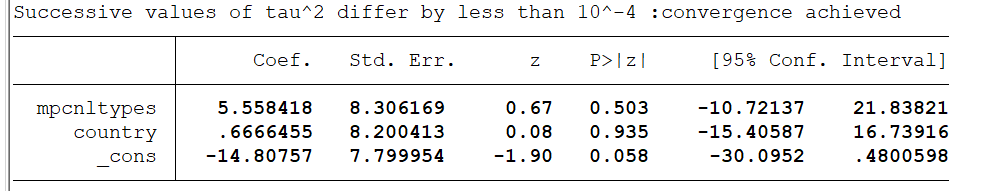


Meta regression of hospitalization time


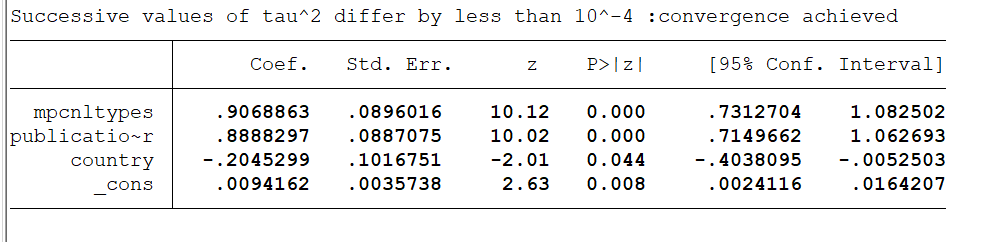


Meta regression of pain visual analogue score


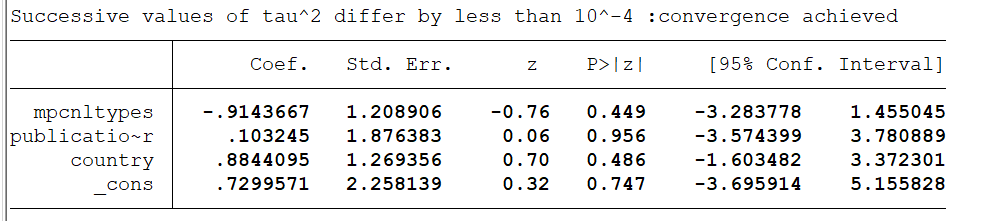


Meta regression of hb drop


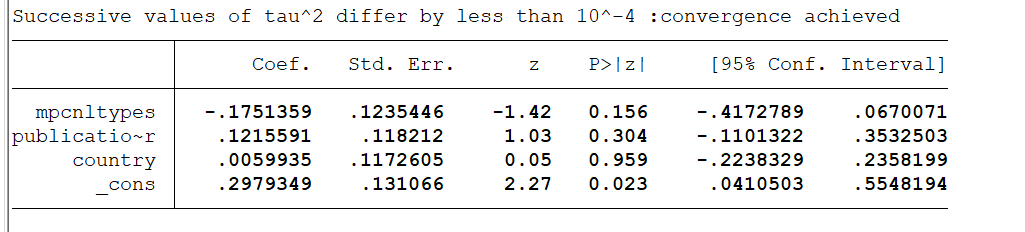

Supplement: Supplementary file 1 — Additional file 1. [file 12894_2023_1341_MOESM1_ESM.docx]
