# Supplementary material for: Efficacy and safety of minimally invasive percutaneous nephrolithotomy versus retrograde intrarenal surgery in the treatment of upper urinary tract stones (> 1 cm): a systematic review and meta-analysis of 18 randomized controlled trials
Source: BMC Urol. 2023 Oct 24;23:171. doi: 10.1186/s12894-023-01341-3 (PMC10598962; doi:10.1186/s12894-023-01341-3)

Subgroup analysis of SFR based on mPCNL type


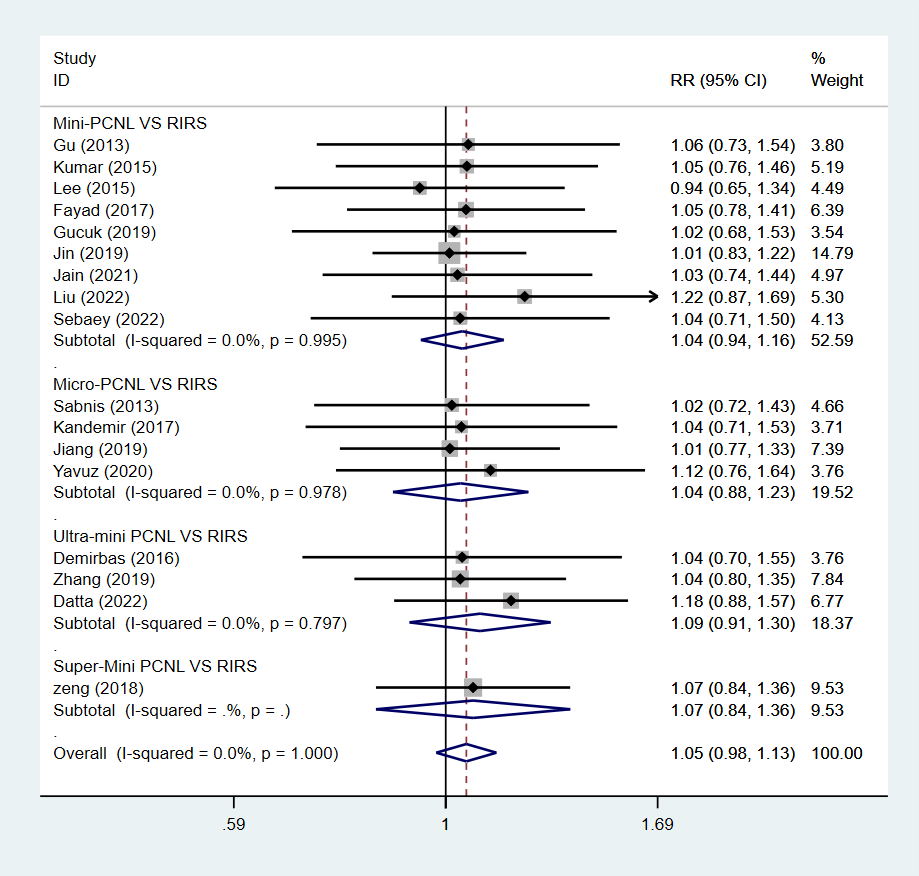


Subgroup analysis of operative time based on mPCNL type
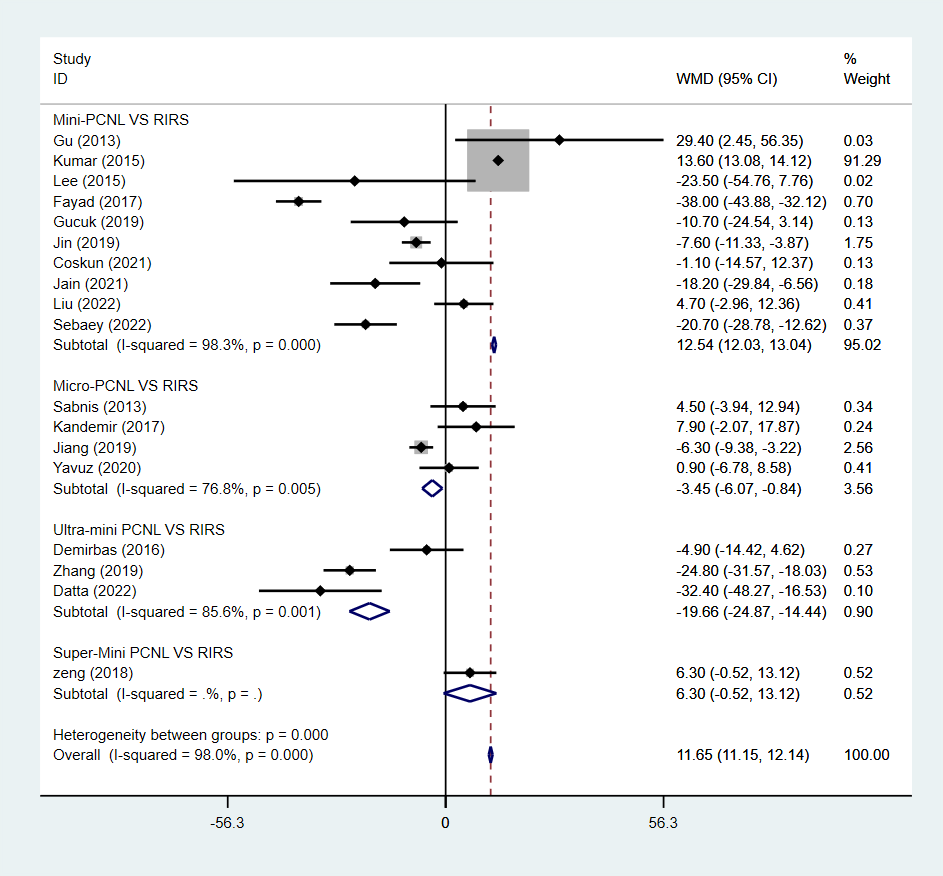


Subgroup analysis of hospitalization time based on mPCNL type


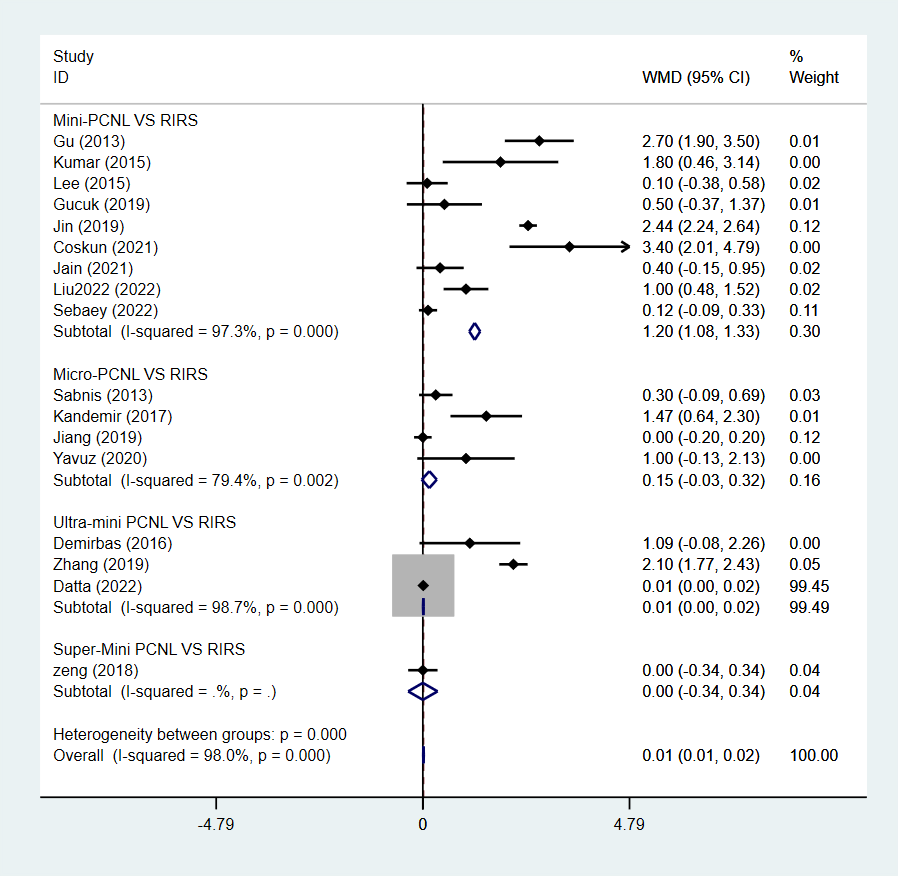


Subgroup analysis of Hb drop based on mPCNL type


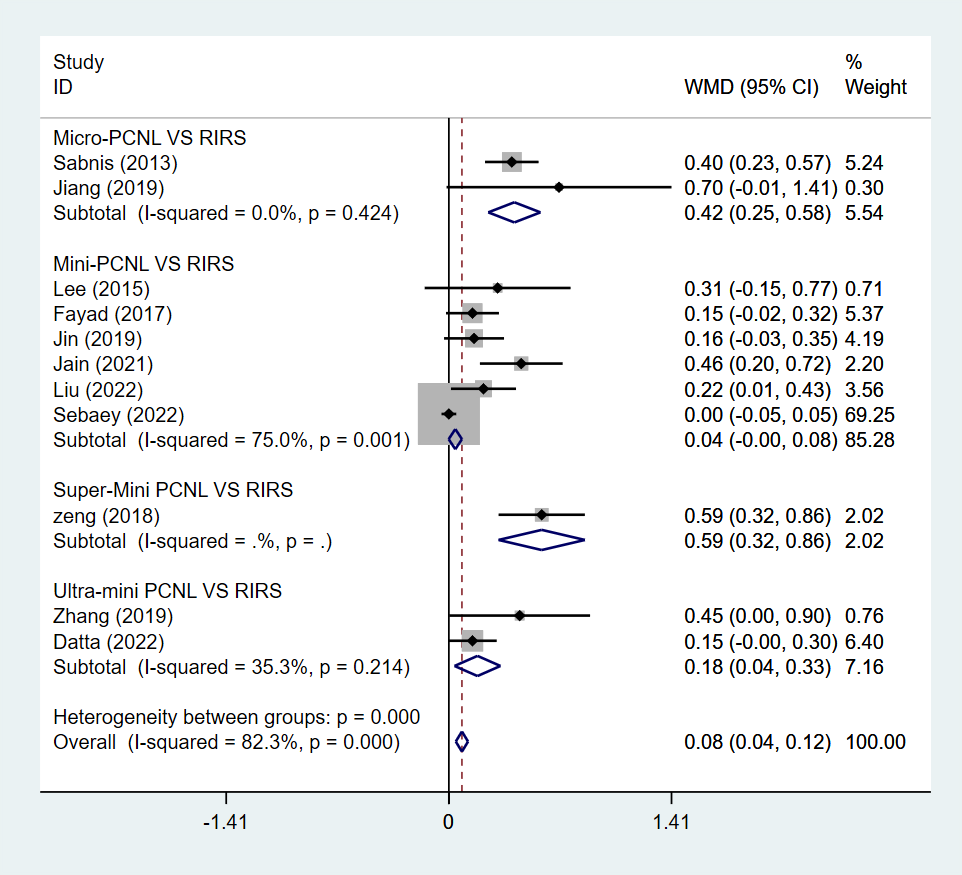


Subgroup analysis of complication ratio based on mPCNL type


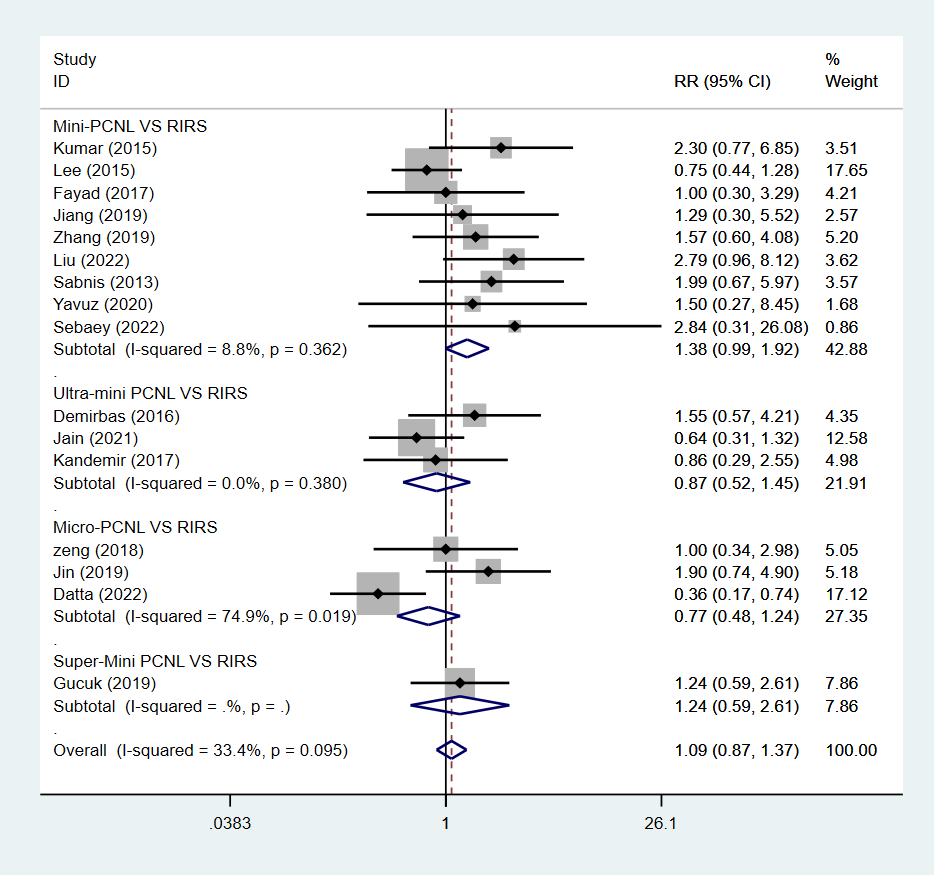


Subgroup analysis of Clavien-Dindo（I-II）based on mPCNL type


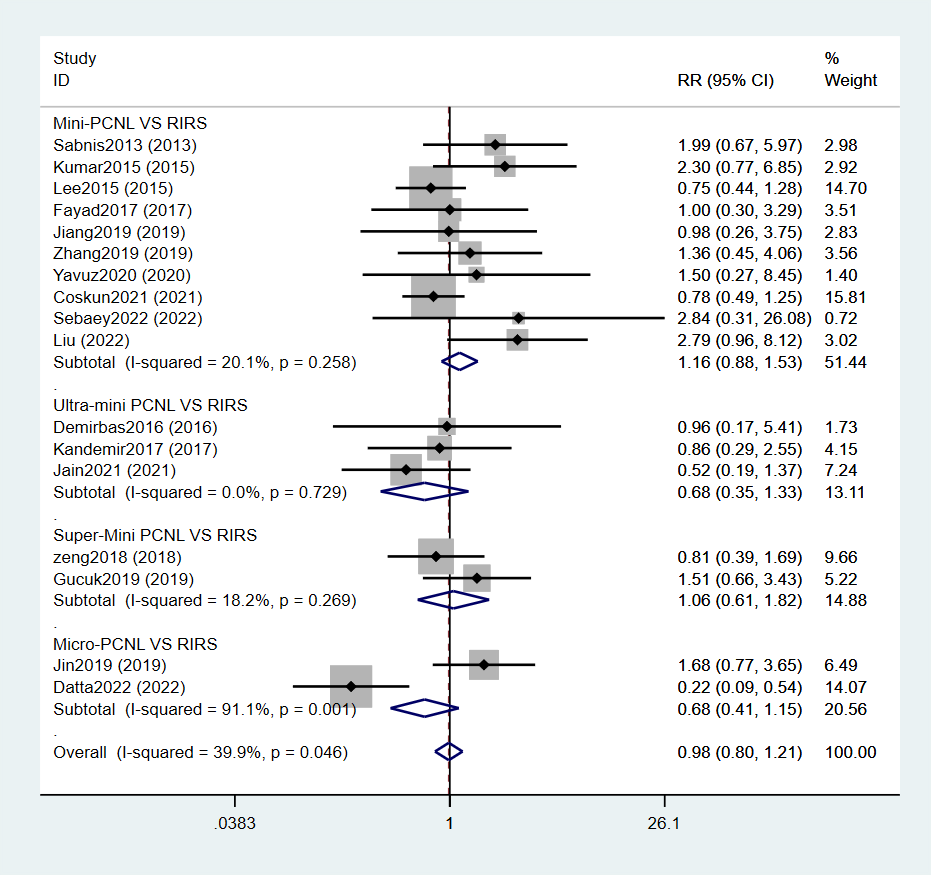

Supplement: Supplementary file 2 — Additional file 2. [file 12894_2023_1341_MOESM2_ESM.docx]
